# Effects of Top and Bottom Electrodes Materials and Operating Ambiance on the Characteristics of MgF_x_ Based Bipolar RRAMs

**DOI:** 10.3390/nano13061127

**Published:** 2023-03-22

**Authors:** Nayan C. Das, Yong-Pyo Kim, Sung-Min Hong, Jae-Hyung Jang

**Affiliations:** 1 Department of Energy Engineering, Korea Institute of Energy Technology, Naju 58330, Republic of Korea; 2 School of Electrical Engineering and Computer Science, Gwangju Institute of Science and Technology, Gwangju 61005, Republic of Korea

**Keywords:** RRAM, electrode materials, operating ambiance, forming-free, surface roughness

## Abstract

The effects of electrode materials (top and bottom) and the operating ambiances (open-air and vacuum) on the MgF_x_-based resistive random-access memory (RRAM) devices are studied. Experiment results show that the device’s performance and stability depend on the difference between the top and bottom electrodes’ work functions. Devices are robust in both environments if the work function difference between the bottom and top electrodes is greater than or equal to 0.70 eV. The operating environment-independent device performance depends on the surface roughness of the bottom electrode materials. Reducing the bottom electrodes’ surface roughness will reduce moisture absorption, minimizing the impact of the operating environment. Ti/MgF_x_/p^+^-Si memory devices with the minimum surface roughness of the p^+^-Si bottom electrode show operating environment-independent electroforming-free stable resistive switching properties. The stable memory devices show promising data retentions of >10^4^ s in both environments with DC endurance properties of more than 100 cycles.

## 1. Introduction

Among the potential non-volatile memory (NVM) technologies, resistive random-access memory (RRAM) is a promising candidate for embedded memory and storage class memory applications due to its excellent scalability, prolonged durability, simple architecture, and compatibility with CMOS technology [1,2,3]. RRAM also has the potential for neuromorphic applications because of its ability to mimic biological synapses [3,4]. Numerous material systems with various switching processes are being studied. Several efforts have been made to design conductive channels to build uniform and controlled conductive filaments in resistive switching devices [1]. However, unstable switching characteristics, including large fluctuations of Set/Reset voltages, are big obstacles to the practical application of RRAM [1,5,6].

Numerous models have been developed to improve RRAM’s uniform switching behaviors, such as structure optimization, metal ion transplanting, adding a metal interface layer, metal doping, electrode optimization, and interface engineering [1,2,3]. RRAM devices with varied electrode materials and the same oxide layer have been reported to exhibit different electrical properties [3,7,8]. The electrodes in RRAM devices significantly impact how resistive switching (RS) behaves. Due to the variations in the work functions of various electrodes and the type of contact between an electrode and an active layer, electrode materials influence distinct RS behaviors by changing the barrier height at the electrode/active layer interface [6,7,9,10,11,12].

Materials selection requires understanding the material characteristics influencing each device’s performance factors, such as on/off ratio, switching speed, retention time, and durability. It is also essential to understand how the operating environment affects the performance of the RRAM device to develop and control its properties [13]. The electrode metals and operating conditions affect the vacancy formation energy [14]. In oxygen-rich environments, the heat of formation for each oxygen atom in the bulk oxide tends to be equivalent to the vacancy formation energy. In contrast, it tends to be near zero in oxygen-poor environments [14]. Additionally, when the device size reduces, the impacts of gaseous ambiance become more pronounced due to the larger specific surface area. Moreover, surface roughness affects a device’s performance in the open air and under a vacuum because it increases moisture absorption [10,15,16,17].

Binary oxide-based materials have recently undergone in-depth research in a vacuum as an active layer of RRAM devices [18,19,20,21,22]. Due to the abundance of oxygen in the atmosphere, the performance of the memory devices and the oxygen vacancy-based active layer are greatly influenced by the working environment. According to studies, most binary oxide-based devices cannot be electroformed and are unstable in a vacuum [18,19,20,21,22]. In a vacuum, one way to overcome the limitations of oxygen vacancy-based RRAMs is to investigate alternate anion vacancy-based materials, which are less affected by the working environment.

Our latest work demonstrates the fluoride vacancy-based bipolar RS characteristics of Ti/MgF_x_/Pt devices in an open-air environment and a vacuum [23,24,25]. However, no research has yet been conducted on the effects of electrode materials (top and bottom) and operating environments (open-air and vacuum) on the performance of the fluoride vacancy-based device.

In this study, we have investigated the effects of electrode materials (top: Ti, Pt, Au/Ni, ITO, and bottom: Pt, p^+^-Si, n^+^-Si, ITO) on MgF_x_-based RRAM devices. We have also analyzed the effect of the operating environments. The effects of electrode material variations and operating ambiance on device performances can be determined by combining two factors: (1) the work function difference between the bottom and top electrodes (Δ*ϕ*) and (2) the surface roughness of the bottom electrodes. All the MgF_x_-based RRAM devices exhibit bipolar RS characteristics in an open-air and a vacuum environment. However, devices with Δ*ϕ* higher or equal to 0.70 eV are stable in both environments. The effect of the operating environment can be minimized by reducing the surface roughness of the bottom electrodes, which reduces moisture absorption. We have demonstrated operating environment-independent electroforming-free stable Ti/MgF_x_/p^+^-Si memory devices for the first time. Devices maintain similar electroforming-free bipolar RS characteristics in the open air and in a vacuum. Memory devices show promising retention and endurance properties.

## 2. Materials and Methods

E-beam evaporation was used to deposit a 150-nm-thick Pt bottom electrode on SiO_2_/Si substrate to fabricate Ti/MgF_x_/Pt devices. A circular shadow mask with a radius of 50 μm was used to design the different top electrodes (TE) (Ti = 150 nm, Pt = 150 nm, Au/Ni = 100/40 nm, ITO = 150 nm). As a bottom electrode (BE) variation, Pt was replaced by p^+^-Si, n^+^-Si, and ITO-coated glass.

The electrical characteristics of the memory devices were measured using a semiconductor parameter analyzer (HP-4155A) in a laboratory atmospheric ambiance. The top electrode received direct voltage, while the bottom electrode was grounded. The electrical characteristics of RRAM devices in a vacuum environment were measured using the MS-TECH Vacuum Chamber Probe Station (10^−3^ torr).

At least three batches of samples for each device type were analyzed to ensure reproducibility. A batch consists of more than twenty devices. More than fifty devices were measured at each condition to confirm the observations and conclusions. Because few process variables were involved in device fabrication and each process condition was well controlled, the range of device-to-device variation was smaller than the range of cycle-to-cycle variation.

## 3. Results and Discussion

In the previous works, the characterization of the MgF_x_ thin films and the performance of Ti/MgF_x_/Pt devices were explored in a laboratory atmospheric ambiance and a vacuum [23,24,25]. The XRD pattern, SEM image, XPS analysis, and FTIR absorbance spectroscopy measurement results for the MgF_x_ thin film, shown in the Supplementary File in Figure S1, are used to thoroughly study the structural, elemental, and compositional properties of the MgF_x_ thin film. A summary of the MgF_x_ thin films characterization is given below.

With an Mg/F ratio of about 1:1.65, which indicates fluoride vacancies in the film, the amorphous defect-rich granular-structured MgF_x_ layer was deposited [23,25]. In open-air environemts, several weak hydroxyl groups and CO_2_ absorption peaks have been observed. These hydroxyl groups show that moisture was absorbed from the environment during the manufacturing or measuring processes and was present on the surface of amorphous MgF_x_. The hydroxyl groups weakly attached to the amorphous MgF_x_ thin film surface are easily removed in a vacuum environment. These loosely connected groups affect the characteristics of the Ti/MgF_x_ interface and the amorphous MgFx active layer. Consequently, the operating environment impacts the device’s performance [25].

Devices with six different electrode materials (Ti, Au/Ni, Pt, ITO, p^+^-Si, and n^+^-Si) were fabricated. The effects of top and bottom electrode materials and operating environment on the MgF_x_-based RRAM devices’ performance are systematically explored as follows:

### 3.1. Effect of Top Electrodes and Operating Environment

**Figure 1:**
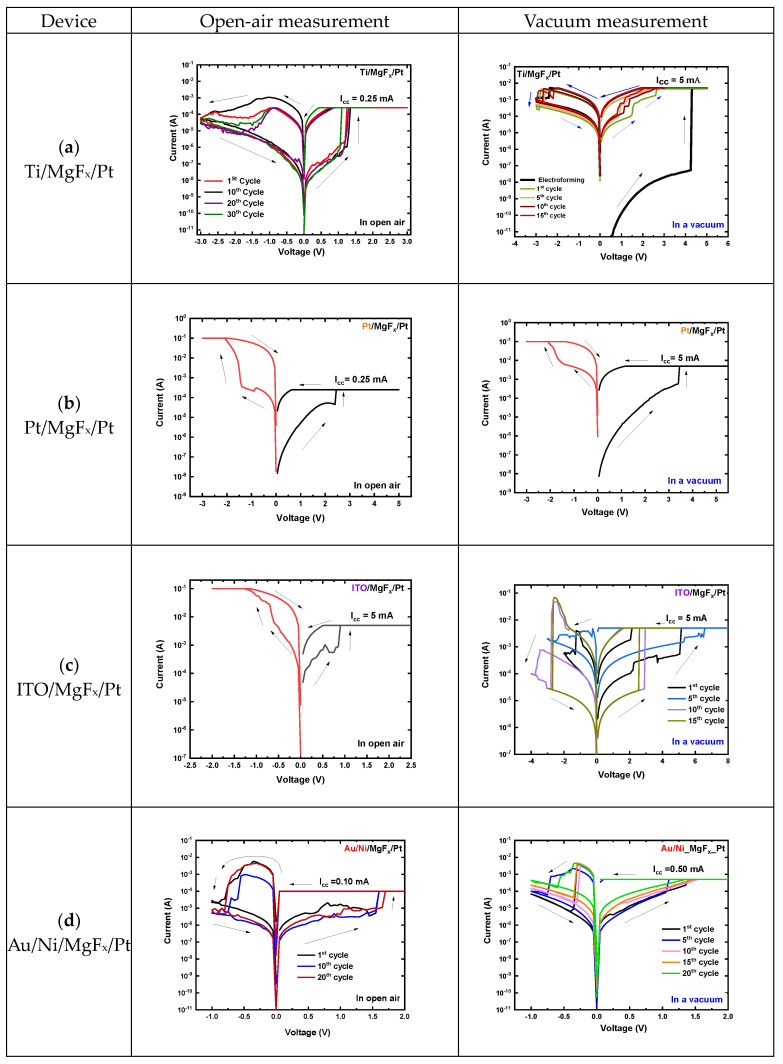
Typical I-V characteristics of MgF_x_-based RRAM devices in open-air environment and vacuum. (**a**) Ti/MgF_x_/Pt (**b**) Pt/MgF_x_/Pt, (**c**) ITO/MgF_x_/Pt, and (**d**) Au/Ni/MgF_x_/Pt.

The RS characteristics of MgF_x_-based RRAM devices with TE (Ti, Pt, Au/Ni, or ITO) and BE (Pt) are measured in open-air and vacuum environments, as shown in Figure 1.

In an open-air environment, by applying a double voltage sweep in the sequence of 0 V → +3 V → 0 V → −3 V → 0 V with the compliance current (I_cc_) of 0.25 mA, electroforming free bipolar RS behavior of the Ti/MgF_x_/Pt devices was observed with an on/off ratio > 10^2^.

In a vacuum environment, the RS features of a device must be activated using an electroforming process, where the initial resistance is higher than in an open-air environment (Figure 1a). In a vacuum, the following changes occur in device performance: (1) SET voltage drops from 1.25 V to 1.0 V, (2) RESET voltage changes from −0.9 V to −2.5 V, (3) SET and RESET current rise, and (4) on/off ratio falls from over 10^3^ to 10 [23,25].

The Pt/MgF_x_/Pt devices show one-time RS from initial HRS to LRS and breakdown during negative bias voltage in the open-air environment and vacuum (Figure 1b). This phenomenon is attributed to the electroforming process, which forms irreversible conduction paths at the electrode–film interface [26].

The ITO/MgF_x_/Pt devices show interesting responses to the operating environment. In the open-air setting, devices do not show repetitive RS properties. However, RS properties are confirmed in a vacuum with high fluctuations (Figure 1c). SET voltage varies from 2.25 V to 6.5 V, and the RESET voltage varies from −0.5 V to −2.5 V, with different ranges of bias voltages.

The Au/Ni/MgF_x_/Pt devices show electroforming-free RS properties in the open-air environment and vacuum with 0 V → +2 V → 0 V → −1 V → 0 V bias voltage and an on/off ratio from over 10^2^ with different compliance currents (Figure 1d). The SET voltage varies from 1.0 V to 1.5 V, and the RESET voltage varies from −0.5 V to −0.75 V. Devices show good potential to be free from operating environmental effects. However, devices are not stable. After around 20 cycles, devices break down.

### 3.2. Effect of Bottom Electrodes and Operating Environment

**Figure 2:**
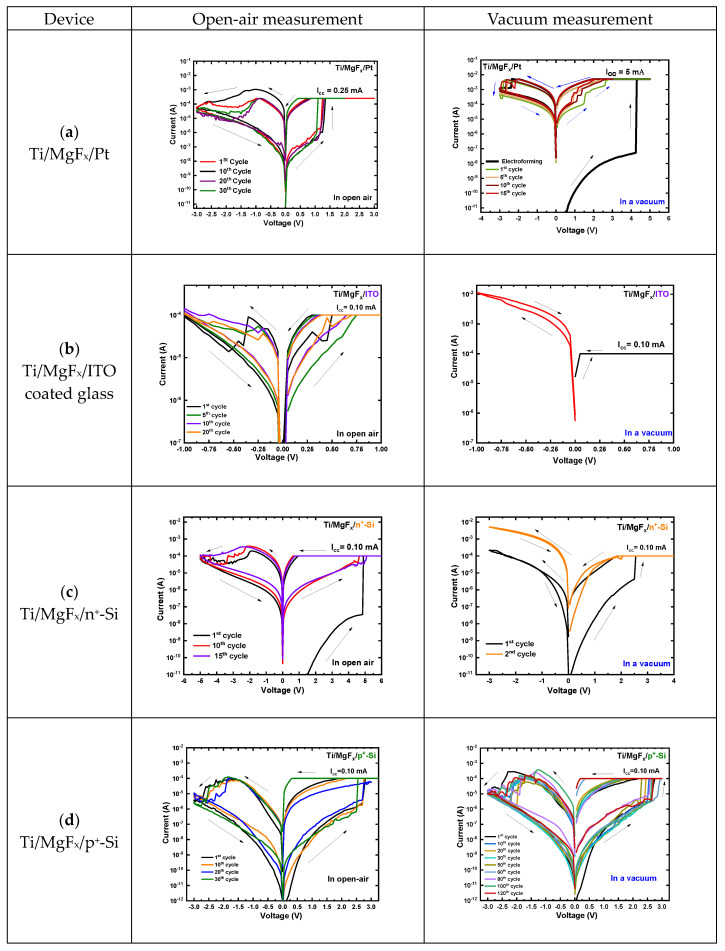
Typical I-V characteristics of in open-air and vacuum. (**a**) Ti/MgF_x_/Pt (**b**) Ti/MgF_x_/ITO, (**c**) Ti/MgF_x_/n^+^-Si, and (**d**) Ti/MgF_x_/p^+^-Si.

After variations of the TE materials in the Ti/MgF_x_/Pt device structure, the BE (Pt) is replaced by ITO-coated glass, n^+^-Si, or p^+^-Si substrates, keeping Ti as the top electrode. The I-V characteristics of MgF_x_-based RRAM with different bottom electrodes in the open-air environment and vacuum are shown in Figure 2.

Ti/MgF_x_/ITO devices also show electroforming-free RS characteristics in the open-air environment but fail to retain RS properties in the vacuum environment (Figure 2b). In an open-air environment, the SET voltage varies from 0.35 V to 0.75 V; the RESET voltage varies from −0.25 V to −0.50 V under the I_cc_ of 0.10 mA, and the voltage sweep range is +1 V to −1 V. However, in a vacuum environment, the device becomes very conductive. This conductivity can be attributed to the O^2−^ escape from the ITO bottom electrode. The formation of oxygen gas in a vacuum environment makes the device more conducive to oxygen vacancies [27].

Ti/MgF_x_/n^+^-Si devices exhibit uniform RS properties in the open air, with a voltage sweep range of +6 V to −5 V and an I_cc_ of 0.10 mA. The SET voltage ranges from 4.5 V to 5.25 V, and the RESET voltage varies from −2.0 V to −2.50 V. However, devices do not show stable RS properties in a vacuum (Figure 2c).

Only Ti/MgF_x_/p^+^-Si devices are less affected by the changing operating environment and show stable electroforming-free RS properties both in open-air conditions and the vacuum. A readout voltage (V_Read_) was +0.50 V. In both open-air and vacuum environments, the SET voltage ranges from 2.25 V to 2.75 V, and the RESET voltage varies from −1.75 V to −2.25 V. With the voltage sweep range +3 V to −3 V, the on/off ratio is >10^2^ in an open-air environment and a vacuum (Figure 2d).

### 3.3. Factors to Determine the Effect of Electrodes and Operating Environment

The effects of electrode materials variations and operating ambiance on device performances can be determined by combining two factors: (1) the work function difference between the bottom and top electrodes [9,10,11,12] and (2) the surface roughness of the bottom electrodes [10,15,16,17]. The work functions of the electrode materials are shown in Table 1, and the work function difference between the TE and BE of devices is shown in Table 2 [6,28].

The difference in work functions of BE and TE materials determines the electric field across the MgFx switching layer in a thermal equilibrium with no electrical bias [10]. From the device performance and the work function difference (Δ*ϕ*) between TE and BE, it is identified that the Δ*ϕ* should be higher or equal to 0.70 eV for stable MgF_x_-based RRAM devices in open-air and vacuum environments. When the Δ*ϕ* is smaller than 0.70 eV, RS properties are unstable and depend on the environment.

**Figure 3:**
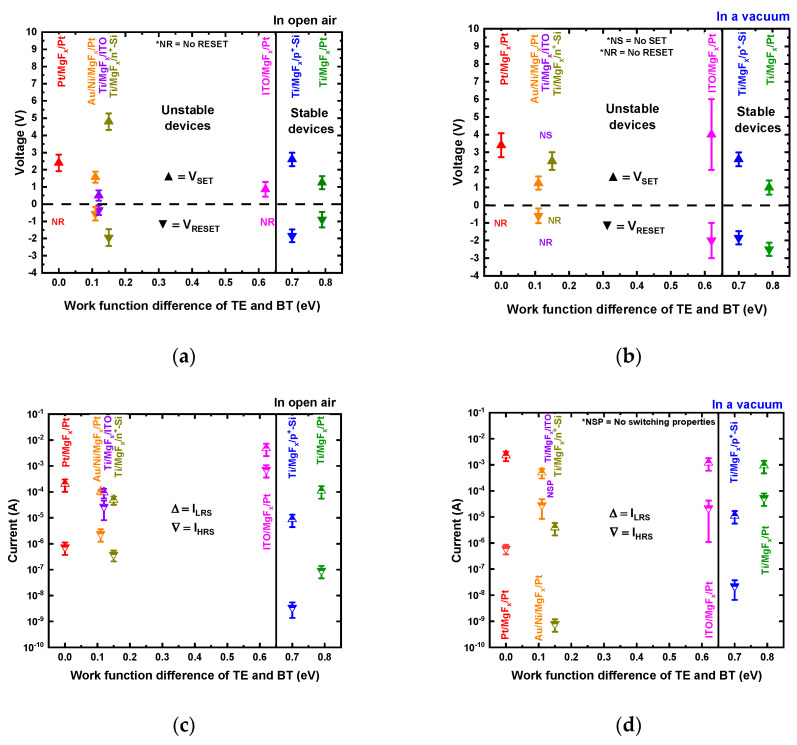
The devices properties with work function differences of TE and BE. (**a**) V_SET_ and V_RESET_ in open air, (**b**) V_SET_ and V_RESET_ in a vacuum, (**c**) I_LRS_ and I_HRS_ in open air, and (**d**) I_LRS_ and I_HRS_ in a vacuum.

Figure 3 summarizes the device properties (V_SET_, V_RESET_, I_LRS_, and I_HRS_) with work function differences of TE and BE in open-air (Figure 3a,c) and vacuum environments (Figure 3b,d). The acronyms used to represent device properties properly in the graphs are NR (No RESET), NS (No SET), and NSP (No switching properties). A device is considered stable if it shows DC endurance properties over 100 cycles and more than 10^4^ s of data retention. Any device that fails to meet the criteria is considered unstable.

The experimental results show that in any given environment (open-air and vacuum), out of the seven kinds of MgF_x_-based devices (Ti/MgF_x_/Pt, Pt/MgF_x_/Pt, ITO/MgF_x_/Pt, Au/Ni/MgF_x_/Pt, Ti/MgF_x_/ITO, Ti/MgF_x_/n^+^-Si, and Ti/MgF_x_/p^+^-Si), two types of devices (Ti/MgF_x_/Pt and Ti/MgF_x_/p^+^-Si) are very stable. Ti/MgF_x_/Pt memory devices’ stability (retention and endurance) in the open air and a vacuum environment are shown in previous works [23,24,25]. Ti/MgF_x_/p^+^-Si memory devices’ DC endurance properties are shown in Figure 2d, showing more than 120 cycles in a vacuum.

**Figure 4:**
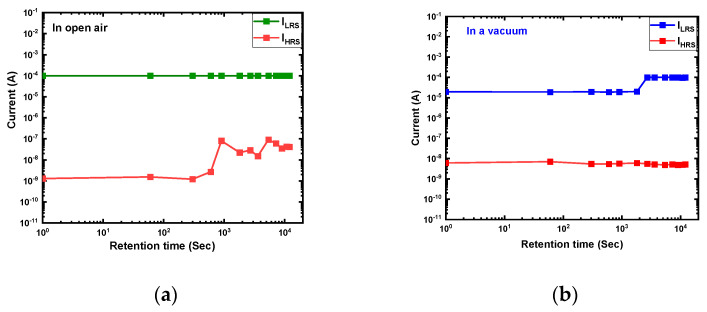
Data retention characteristics of Ti/MgF_x_/p^+^-Si memory devices. (**a**) In open-air environment; (**b**) in a vacuum environment.

The data retention properties of the Ti/MgF_x_/p^+^-Si memory devices in the open air and a vacuum environment are shown in Figure 4. In both operating environments, the device exhibits good data retention over 10^4^ s with an on/off ratio greater than 10^3^. The LRS state is more stable and uniform in open-air measurements than the HRS. In a vacuum environment, both states are comparatively stable with time.

An e-beam deposited BE, and TE surface is significantly more uneven than a Si wafer surface as BE. The BE’s surface roughness significantly influences the device’s stability and the operational environment’s impact [10,15,16,17]. The MIM structure device’s rough surface can create more traps between the electrodes and the active layer at the interface. Furthermore, the degree of BE roughness significantly affects RS. The switching voltages are impacted by the local field-concentrating regions of the surface [10,15,16,17]. Additionally, surface roughness enhances moisture absorption, affecting the device’s performance in open-air and vacuum environments [17].

In an open-air environment, the pristine MgF_x_-based devices contain significant internal and external defects. The internal defects are fluoride vacancies in the bulk MgFx active layer, and the external defects are weakly bound to O-H groups on the surface of MgF_x_. These external defects enable the dissociation of O^2−^ and H^+^ ions to generate anion vacancies at the interface. As a result, the ionic charge carriers in the interface region differ from those in bulk MgF_x_, increasing the conductivity of the interface region [22,29,30]. As a result, most of the MgF_x_-based devices show electroforming-free RS properties in an open-air environment.

However, weakly bound O-H groups and CO_2_ are eliminated from the interface region in a vacuum environment. Thus, only an electronic current is present, and the ionic charge carriers (O^2−^ and H^+^) are gone. As a result, the active layer becomes more resistant, increasing the devices’ overall initial resistance, and an electroforming process is necessary to activate its RS capabilities [18,19,20,21,22,23,24,25,31].

The small Δ*ϕ* and surface roughness can explain the devices’ instability. The surface roughness values of substrates and BEs are presented in Table 3. It is easy to make a conduction filament between TE and the active layer without an electroforming process in an open-air environment because of the small Δ*ϕ* and existence of external defects (O-H groups) caused by the roughness of the BE and active layer surface [23,25]. As a result, most of the combinations of MgF_x_-based devices show electroforming-free RS properties. However, within a few cycles, a permanent conduction filament is formed between TE and the active layer, and the device loses RS properties. The fluctuation Ti/MgF_x_/ITO in a vacuum can be attributed to the ITO-coated glass’s highest surface roughness (SR_RMS_ = 4.050 nm).

From the different top and bottom electrode combinations of MgF_x_-based devices, Ti/MgF_x_/Pt and Ti/MgF_x_/p^+^-Si are the most stable. However, the Ti/MgF_x_/p^+^-Si device is less affected by the operating environment and shows very stable electroforming-free RS performance in open-air and vacuum environments.

**Figure 5:**
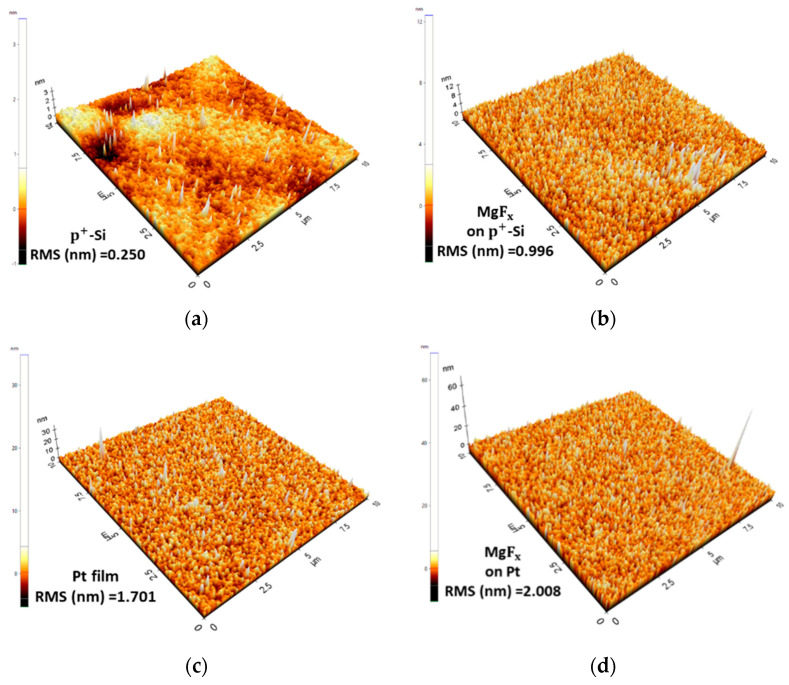
Surface roughness (**a**) the p^+^-Si, (**b**) MgF_x_ on p^+^-Si (**c**) Pt, (**d**) MgF_x_ on Pt. Area is 10 μm × 10 μm.

The surface morphology of the p^+^-Si, Pt BE, and after the MgF_x_ layer deposition are shown in Figure 5. The p^+^-Si substrate (SR_RMS_ = 0.250 nm) is much smoother than the e-beam deposited Pt BE (SR_RMS_ = 1.701 nm). After MgF_x_ deposition, the surface roughness of the MgF_x_ on the p^+^-Si (SR_RMS_ = 0.996 nm) is much less than that of the MgF_x_ on the Pt (SR_RMS_ = 2.008 nm). Thus, moisture absorption is lower at the interfaces of the Ti/MgF_x_/p^+^-Si device compared to the Ti/MgF_x_/Pt device. As a result, in an open-air environment, the initial resistance of Ti/MgF_x_/p^+^-Si (~GΩ) is higher than that of Ti/MgF_x_/Pt (~10 MΩ), even though p^+^-Si-based devices (0.70 eV) have lower Δ*ϕ* than Pt BE-based devices (0.79 eV).

In a vacuum environment, due to the removal of moisture from the interfaces of the pristine Ti/MgF_x_/Pt devices, the initial resistance (~10 GΩ) of devices is higher than that (~10 MΩ) in an atmospheric environment and needs an electroforming process to activate the RS properties. However, due to the smoother surface and less moisture absorption, Ti/MgF_x_/p^+^-Si devices are less affected by the operating environment and show almost similar performance both in a vacuum and in an open-air environment with the same initial resistance (~GΩ), SET, and RESET voltages.

### 3.4. Conduction and RS Mechanism of Ti/MgF_x_/p^+^-Si Devices

**Figure 6:**
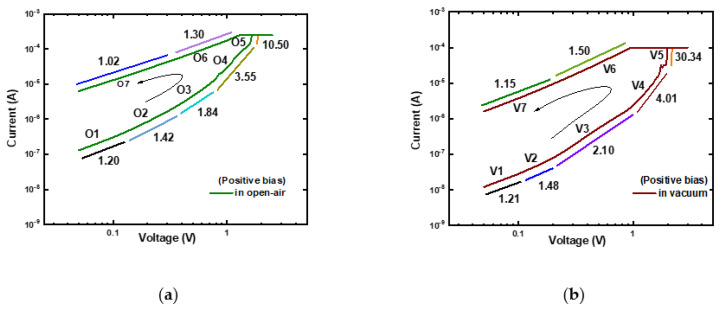
Log (I)–log (V) characteristics of Ti/MgF_x_/p^+^-Si memory devices with slopes of different parts. (**a**) In an open-air environment; (**b**) in a vacuum environment.

Forward bias regions of typical I–V curves are replotted as log(I)−log(V) to explore the conduction mechanism of the Ti/MgF_x_/p^+^-Si device in an open-air and a vacuum environment. The curve fittings results are shown in Figure 6. The open-air measurement is divided into O1, O2, O3, O4, O5, O6, and O7, shown in Figure 6a. The vacuum measurement is also divided into V1, V2, V3, V4, V5, V6, and V7, as shown in Figure 6b. Devices show a similar pattern of curve fittings in both open-air and vacuum environments, indicating that the operating environment does not affect the device’s conduction mechanism.

In the open-air measurement, the ohmic conduction (I∝V) was demonstrated at the low positive voltage area by the slope of LRS (O7: 1.02). When the voltage is increased, the slopes of the HRS (O2: 1.42 and O3: 1.84) and LRS (O6: 1.30) follow Child’s rule (I ∝ $V^{n}$ where *n* = 1.3~2). The conduction mechanism at the SET voltage region (O3: 3.55 and O5: 10.5) adheres to Child’s law (I ∝ $V^{n}$ where *n* = 3~11). The slopes (O1: 1.20, V1: 1.21) at the low voltage zones (O1 and V1), however, are marginally higher than 1. The incomplete generation and rupture of CFs during the SET and RESET processes cause the slopes of the fitting lines to be slightly greater than 1 in the lower voltage region [32]. The above analysis and prior studies [23,24] demonstrate that the device’s conduction mechanism is a trap-controlled space charge limited conduction (SCLC), regardless of the operating environment. The RS is driven by the transition from charge trapping and de-trapping to filamentary conduction [6,31,33,34,35].

**Figure 7:**
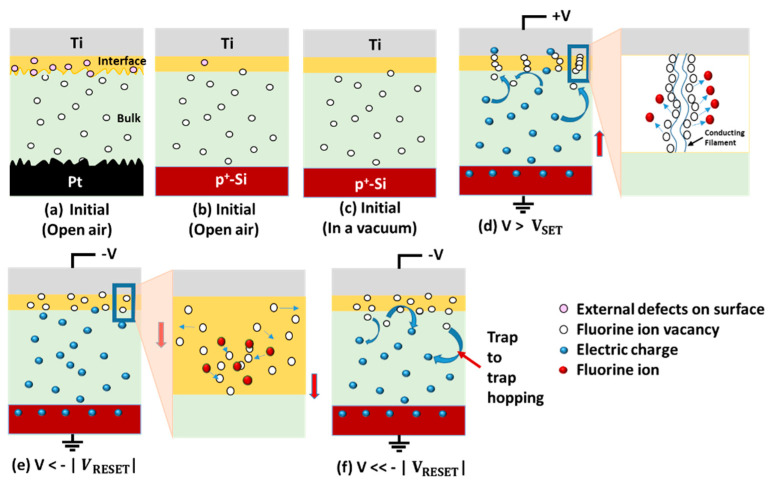
Schematics of proposed switching mechanism of Ti/MgF_x_/p^+^-Si memory device.

The complete resistive switching mechanism with the step-by-step schematics of the Ti/MgF_x_/p^+^-Si memory devices in the open air and a vacuum is proposed and shown in Figure 7. Our previous studies show in detail the area (Ti electrode size variation) and thickness (MgF_x_ layer) independence of MgF_x_-based memory devices [23,24]. These studies indicate that CF-type resistive switching happens at the Ti/MgF_x_ interface of the MgF_x_-based memory devices. The main difference in resistive switching properties between Ti/MgF_x_/Pt and Ti/MgF_x_/p^+^-Si memory devices is the roughness of the bottom electrodes.

All defects (intrinsic and extrinsic) are viewed as traps in MgF_x_-based memory devices. In the bulk MgFx active layer, fluoride (F^−1^) vacancies are regarded as intrinsic defects. Moisture-related defects are considered extrinsic defects. The above analysis shows that the Pt BE is significantly rougher than the p^+^-Si substrate. As a result, compared to the MgF_x_ on the Pt, the surface roughness of the p^+^-Si is significantly lower. Thus, compared to a Ti/MgF_x_/Pt device (Figure 7a), the Ti/MgF_x_/p^+^-Si (Figure 7b) absorbs less moisture at the interface [10,15,16,17]. In a vacuum environment, moisture-related extrinsic defects are removed from the interfaces. However, Ti/MgF_x_/p^+^-Si devices are less impacted by the operating environment and exhibit nearly identical initial states in both a vacuum and an open-air environment because of the smoother surface and lower moisture absorption in the latter (Figure 7c).

When a positive bias voltage is applied, the injection of electrons in the lower voltage region is relatively small. It is primarily dominated by free carriers produced thermally inside the MgF_x_ film due to the p^+^-Si BT. The injection of electrons increases as the bias voltage rises. As a result, the injected electron concentration gradually exceeds the film’s equilibrium electron concentration and controls the device’s current conduction. The injected electrons are partially trapped by the traps in the bulk MgF_x_ film when the voltage approaches near V_SET_. As a result, the charge-trapping mechanism makes conduction routes from the bottom electrode to the interface through the traps in the MgF_x_ layer’s bulk (Figure 7d). At V_SET_, fluoride vacancies-based CF is formed in the interfacial region of Ti/MgF_x_ layers (Figure 7d) [36]. In the amorphous MgF_x_ layer, the fluoride vacancies are mostly formed at grain boundaries and are localized at the interface. Due to the applied voltage during the SET process, through grain boundaries, fluoride can accumulate gradually at the electrode and change the potential barrier at electrode/oxide contacts [29,37]. As a result, the resistance state of the device changes from HRS to LRS.

Fluoride ions return to the CF and eventually recombine with the vacancies when a negative voltage is used for the RESET operation. The interface’s CF is partially ruptured at V_RESET_ [38,39]. As a result, the interface region turns resistive, and the total resistance state of the device switches from LRS to a new HRS (Figure 7e). Increasing the negative voltage further, the charge de-trapping mechanism in bulk MgF_x_ reduces conduction routes. From the interface to the bottom, the trapped electrons hop back through the fluoride-related traps of the MgF_x_ layer by employing the SCLC trap-controlled mechanism (Figure 7f) [31,40].

Electroforming-free behavior of the RRAM devices is generally characterized as the result of nonstoichiometric metal oxides, internal defects, and CF confinement [41,42,43]. As Ti/MgF_x_/p^+^-Si memory devices are not much affected by the operating environment, the electroforming-free behavior results from the amorphous defect-rich nonstoichiometric (Mg/F = ~1:1.65) MgF_x_ layer.

## 4. Conclusions

The effects of electrode materials (TE and BE) and the operating environment (open-air and vacuum) on the performance of MgF_x_-based RRAM devices are systematically studied. Experimental results led to two essential findings. First, the device’s performance and stability depend on the difference between the work functions of TE and BE materials. MgF_x_-based RRAM devices with Δ*ϕ* < 0.70 eV show unstable RS or no RS properties depending on the open-air and vacuum environments. With Δ*ϕ* > 0.70 eV, devices offer stable RS properties regardless of the operating environment. Second, the effect of the operating environment on the device performance depends on the surface roughness of the BE. In order to lessen the operating environment effects, the surface roughness of the BEs should be decreased, resulting in less moisture absorption at the interface of Ti/MgF_x_ in the open air.

Ti/MgF_x_/Pt and Ti/MgF_x_/p^+^-Si memory devices show electroforming-free bipolar RS characteristics in the open air. However, Ti/MgF_x_/Pt needs electroforming in a vacuum because removing absorbed moisture makes the device more resistive. In contrast, the Ti/MgF_x_/p^+^-Si device maintains electroforming-free bipolar RS characteristics in a vacuum. The stable memory devices demonstrate encouraging data retention of >10^4^ s with an on/off ratio greater than 10^2^, even after 120 cycles. The operating environment-independent properties of the Ti/MgF_x_/p^+^-Si devices are due to less moisture absorption on the smoother surface of the p^+^-Si substrate. This study moves us one step closer to understanding RRAM performance and improves overall device performance, regardless of the operating environment.

## Figures and Tables

**Table 1:** The work functions of the electrode materials.

Electrode Materials	Ti	Pt	ITO	Au	Ni	n^+^-Si	p^+^-Si
**Work functions (eV)**	4.33	5.12	4.5	5.1	5.01	4.58	5.03

**Table 2:** The work function difference between TE and BE of devices.

Device	Ti MgF_x_ Pt	Pt MgF_x_ Pt	ITO MgF_x_ Pt	Au/Ni MgF_x_ Pt	Ti MgF_x_ ITO	Ti MgF_x_ n^+^-Si	Ti MgF_x_ p^+^-Si
**Work function difference (eV)**	0.79	0	0.62	0.11	0.12	0.15	**0.70**

**Table 3:** Surface roughness of substrate and BEs.

Substrate and BE	SiO_2_	Pt	p^+^-Si	n^+^-Si	ITO Coated Glass
Roughness, RMS (nm)	0.928	1.701	0.250	0.197	4.050

## Data Availability

Not applicable.

## Supplementary Materials

The following supporting information can be downloaded at: https://www.mdpi.com/article/10.3390/nano13061127/s1, Figure S1. Structural and compositional analysis of MgF_x_ thin film (**a**) XRD pattern of MgF_x_ film; (**b**) SEM image of the surface; (**c**) XPS analysis with characteristics peaks and atomic percentages of magnesium and fluorine; (**d**) FTIR absorbance spectra in open air and vacuum environment. References [23,24,25] are cited in Supplementary Materials.
