# Supplementary material for: Effects of Top and Bottom Electrodes Materials and Operating Ambiance on the Characteristics of MgFx Based Bipolar RRAMs
Source: Nanomaterials (Basel). 2023 Mar 22;13(6):1127. doi: 10.3390/nano13061127 (PMC10058438; doi:10.3390/nano13061127)
Supplement: Supplementary file 1 [file nanomaterials-13-01127-s001.zip › nanomaterials-2266530-supplementary.pdf]

Supplementary Materials

Effects of Top and Bottom Electrodes Materials and Operating Ambiance on the Characteristics of MgF_x Based Bipolar RRAMs

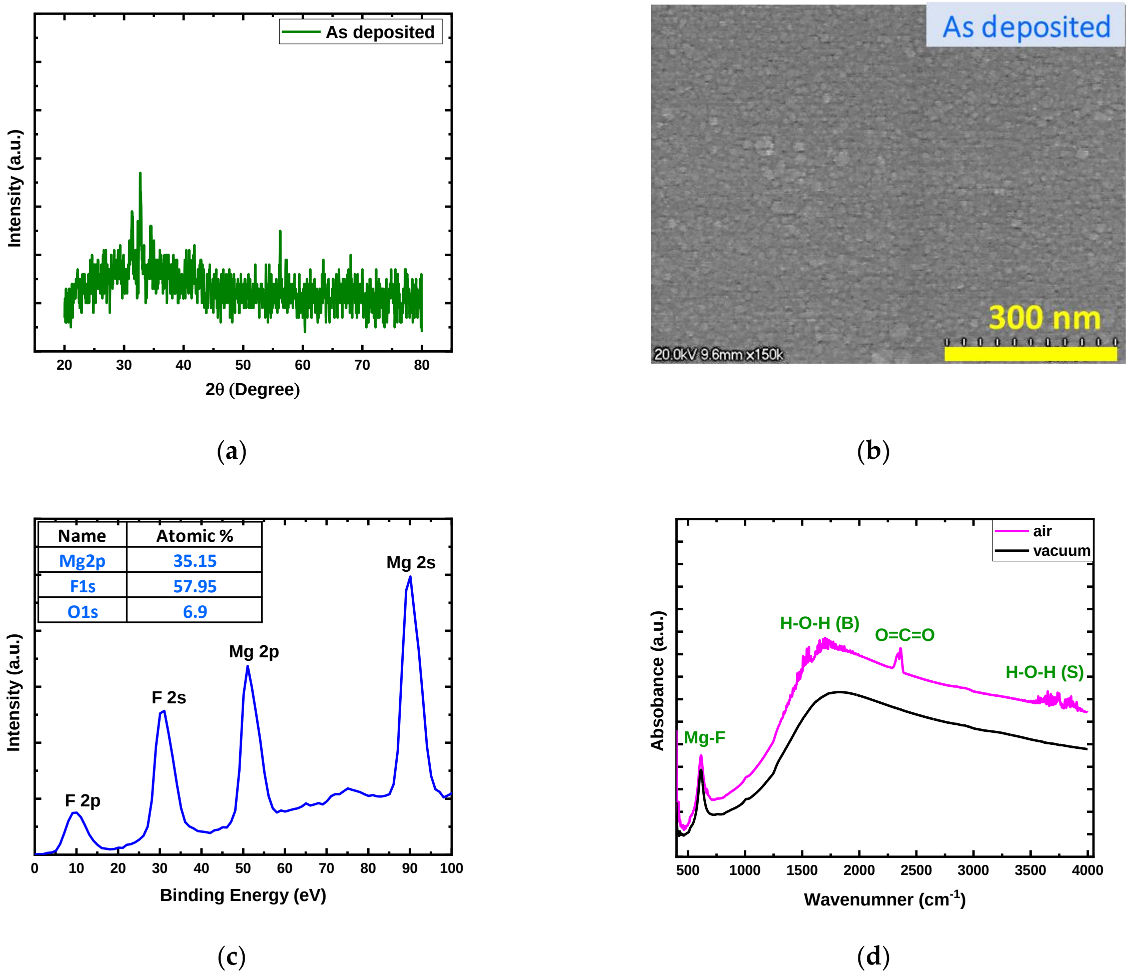

Figure S1. Structural and compositional analysis of MgF_x thin film (**a**) XRD pattern of MgF_x film; (**b**) SEM image of the surface; (**c**) XPS analysis with characteristics peaks and atomic percentages of magnesium and fluorine; (**d**) FTIR absorbance spectra in open air and vacuum environment [23–25].

References

23. Das, N.C.; Kim, M.; Rani, J.R.; Hong, S.-M.; Jang, J.-H. Electroforming-Free Bipolar Resistive Switching Memory Based on Magnesium Fluoride. *Micromachines* **2021**, *12*, 1049, doi:10.3390/mi12091049.
24. Das, N.C.; Kim, M.; Rani, J.R.; Hong, S.-M.; Jang, J.-H. Nanoscale Low-temperature characteristics of magnesium fluoride based bipolar RRAM devices. **2022**, doi:10.1039/d1nr05887h.
25. Das, N.C.; Kim, M.; Kwak, D.U.; Rani, J.R.; Hong, S.M.; Jang, J.H. Effects of the Operating Ambiance and Active Layer Treatments on the Performance of Magnesium Fluoride Based Bipolar RRAM. *Nanomaterials* **2022**, *12*, doi:10.3390/nano12040605.
